# The central regulatory effects of acupuncture in treating primary insomnia: a review

**DOI:** 10.3389/fneur.2024.1406485

**Published:** 2024-12-10

**Authors:** Lin Yao, Yanze Liu, Mengyuan Li, Haizhu Zheng, Mengmeng Sun, Min He, Zhen Zhong, Shiqi Ma, Haipeng Huang, Hongfeng Wang

**Affiliations:** ^1^Institute of Acupuncture and Massage, Northeast Asian Institute of Traditional Chinese Medicine, Changchun University of Chinese Medicine, Changchun, China; ^2^Acupuncture and Tuina Center, The Third Affiliated Clinical Hospital of Changchun University of Chinese Medicine, Changchun, China; ^3^College of Acupuncture and Massage, Changchun University of Chinese Medicine, Changchun, China

**Keywords:** acupuncture, primary insomnia, brain, neural mechanism, neuroimaging

## Abstract

Chronic insomnia has the potential to significantly impact physical well-being, occupational performance, and overall quality of life. This review summarizes the clinical and basic research on the central regulatory mechanism of acupuncture in treating primary insomnia (PI), aiming to explore the clinical effectiveness and possible mechanism of acupuncture in treating PI. The currently available drugs for insomnia exhibit notable adverse effects and tend to induce dependence. Empirical evidence from clinical investigations has demonstrated that acupuncture has a favorable safety profile while substantially enhancing the sleep quality of individuals diagnosed with PI. The combination of acupuncture and medication has been shown to augment the therapeutic efficacy of medication while reducing the dosage and mitigating the occurrence of unwanted effects. A review of the current clinical and basic research on the effects of acupuncture on central alterations in PI patients revealed that acupuncture exerts a regulatory influence on the functional activity of brain regions implicated in cognitive and emotional processes. Additionally, acupuncture has been found to impact metabolite levels and circadian clock gene expression and enhance inflammatory responses and energy metabolism. Notably, a single acupuncture intervention had a modulatory effect on functional brain regions similar to that of cumulative acupuncture. The current clinical trials on acupuncture have been limited in scale, and basic research has focused on a single objective. With the continuous progress of brain research, extensive clinical randomized controlled trials of high quality can be combined with various neuroimaging technology modalities. Moreover, different targets and pathways can be explored through basic research. This may serve to enhance the understanding of the fundamental central nervous system mechanisms involved in the efficacy of acupuncture in treating PI.

## 1 Introduction

According to statistical data, approximately 30–36% of adults have reported experiencing at least one symptom associated with nocturnal sleep disturbances, such as difficulties in falling asleep or maintaining sleep or experiencing irregular sleep patterns (1). Primary insomnia (PI) is a specific type of insomnia that persists even after excluding potential secondary factors. PI is characterized by protracted sleep onset, difficulty maintaining sleep, daytime sleepiness, and impaired cognitive functioning (2). These symptoms significantly impact an individual’s quality of life and work performance (3). Inadequate treatment of insomnia may lead to mental health issues, metabolic illnesses, and cognitive impairment (4). Currently, there is a lack of universal agreement regarding pharmacological interventions for the treatment of insomnia. Four primary categories of medications are commonly employed: GABA sedative hypnotic pharmaceuticals, melatonin agonists, orexin receptor antagonists, and sedative antidepressants. However, patients taking these medications are susceptible to developing tolerance and reliance, which can impact daily functioning in the short term and result in long-term dependency (5). In addition to the use of medication, cognitive behavioral therapy (CBT) is a widely accepted and preferred method for managing chronic insomnia (6–8). However, CBT may also cause side effects or adverse reactions, which may lead to increased daytime sleepiness and a predisposition to accidents or injuries (8, 9). Hence, there has been a continuous endeavor to identify a treatment for PI that is more convenient and has fewer adverse effects.

Acupuncture is an important part of complementary and alternative medicine. Multiple investigations have indicated that acupuncture therapy is highly recommended in neurology, musculoskeletal and connective tissue, obstetrics and gynecology, cancer, gastrointestinal illnesses, and other fields (9–11). At present, many clinical or basic have confirmed that acupuncture has benign regulatory effects on sleep quality, especially for primary insomnia, depression-related insomnia, and cancer-related insomnia (12, 13), with few adverse reactions and low costs (14). Clinical studies have shown that acupuncture significantly improves subjective parameters such as sleep quality and duration compared with sham acupuncture or drug therapy (15). The objective outcome indices of acupuncture for improving the severity of insomnia are also better than those of sham acupuncture, which are shown to significantly improve sleep efficiency and total sleep time, as measured by polysomnography and an activity recorder (16, 17). Basic scientific research in animal models shows that acupuncture can stimulate neurons in the brain (18); affect the synthesis, release and role of sleep-related neurotransmitters (such as catecholamine, glutamic acid, and melatonin) (19–22); increase the nitric oxide content in the brain and blood (23); and regulate the neural activity of the autonomic nervous system (24), thus providing a biological rationale for the treatment of insomnia. With the wide application of acupuncture in the treatment of insomnia, many studies have explored their clinical efficacy and potential central mechanism. Recent studies have also clarified the key position of autonomic nerves in the therapeutic role of acupuncture (25). Due to the establishment of the brain plan, the application of central nervous system imaging technology in the clinical research of acupuncture in treating PI patients has increased (26, 27). Neuroimaging technology can be used to visually recognize changes in the central nervous system (28). This approach helps explore the regulatory role of acupuncture in abnormal functional networks. Several studies have used neuroimaging techniques to assess the clinical impact of acupuncture in treating PI patients. Research shows that acupuncture can regulate the function or irregularity of many regions in the brain (29, 30). However, the existing research has not yet explored the exact central mechanism of acupuncture to improve sleep quality, which to some extent limit the clinical application of acupuncture in treating PI, and may cause patients to miss timely and effective treatment.

In view of the increasing literature on the effectiveness of acupuncture in treatment PI and neurobiological pathways, there is still a lack of comprehensive review on the central brain regulation mechanism of acupuncture in treatment PI. Therefore, it is now time to synthesize and review the existing evidence of the mechanism and clinical effectiveness of acupuncture in treatment PI, aiming to consolidate its effectiveness and guide future research, and provide a new perspective on the clinical use of acupuncture for enhancing sleep quality and regulating the sleep–wake cycle in individuals with PI.

## 2 Methods

### 2.1 Search strategy

**Figure 1:**
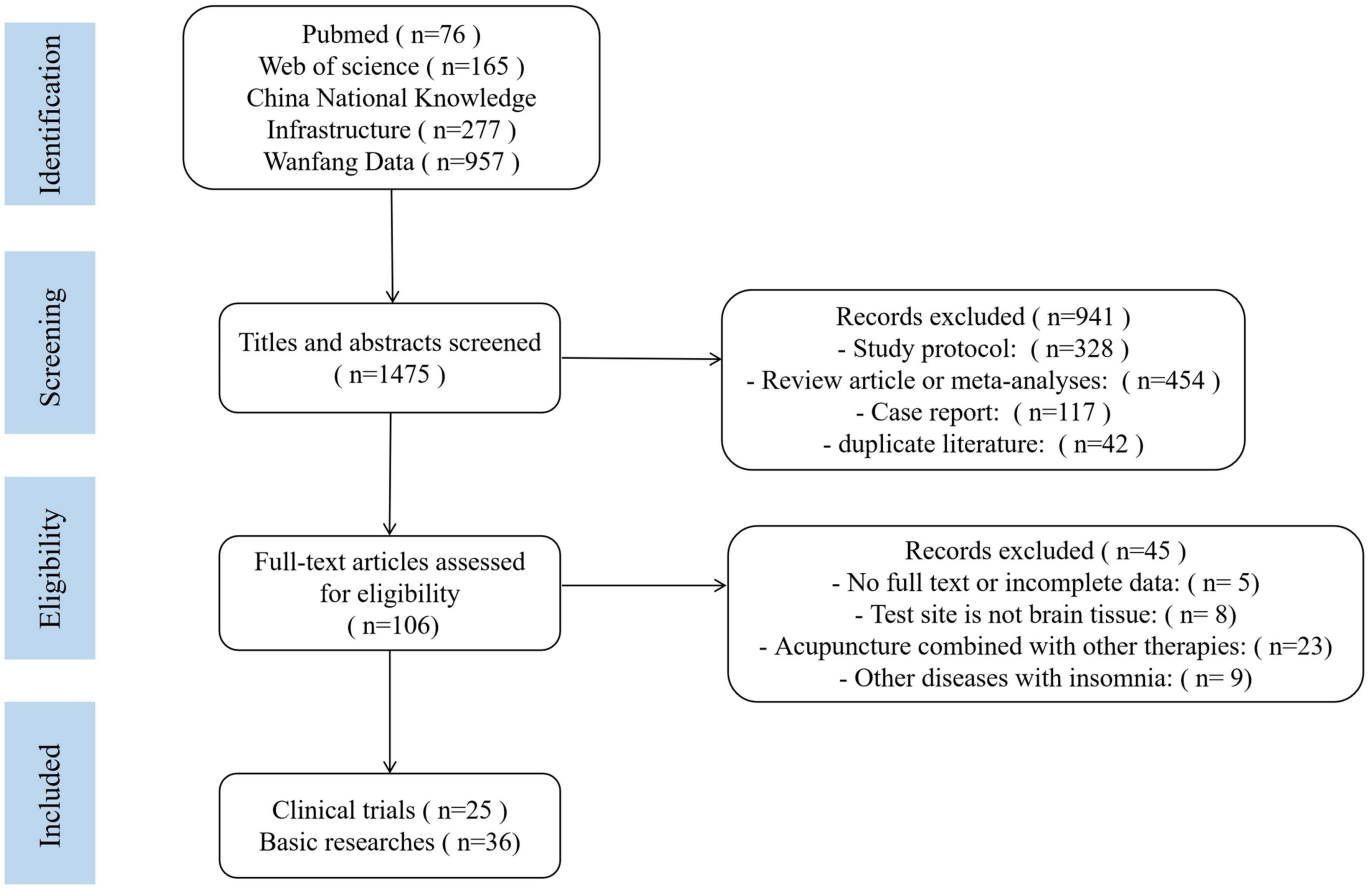
The flow diagram of literature screening.

The following six electronic databases have been accessed since the database was created until June 2024 with no restrictions on the state of publications: PubMed, web of science, China National Knowledge Infrastructure and Wanfang Data. Relevant articles from references and reviews of selected publications were also verified. Search terms included “acupuncture” and “insomnia.” After initial screening, a total of 1,475 articles were retrieved. The retrieved studies were filtered using Zotero, and 941 articles were filtered based on title and abstract. Another 45 articles were filtered by reading the full text, and 25 clinical trials and 36 basic research articles were finally included. The flow diagram of literature screening is shown in Figure 1.

### 2.2 The inclusion and exclusion criteria

1. Research types. This review includes all published clinical trials and animal basic researches on acupuncture treatment of primary insomnia. The language is limited to English and Chinese. Exclude meta-analyses, reviews, experimental protocols, cases, conference papers, and dissertations. At the same time, studies without full-text or incomplete data should be excluded.
2. Research participants. Primary insomnia patients (as diagnosed by a clinician, or using any recognized diagnostic criteria). Exclude participants with other severe mental disorders or physical illnesses, or patients with insomnia due to other illnesses.
3. Intervention methods. Manual acupuncture, electroacupuncture, transcutaneous auricular vagus nerve stimulation, auricular acupuncture, warm acupuncture or shallow acupuncture. Exclude studies that combine other treatments.
4. Research results. The study explored the regulation results of acupuncture on the brain center, and excluded studies that did not involve the regulation of the brain center.

### 2.3 Data extraction

The purpose of the literature search was to analyze the mechanisms of central brain regulation of PI by acupuncture. Validate the final included articles and extract relevant data. Any disagreements were resolved by discussions between authors.

## 3 Clinical efficacy of acupuncture in treating PI

The number of studies about the efficacy and safety of acupuncture as a treatment modality for insomnia is experiencing notable growth. Multiple systematic reviews and meta-analyses provide empirical evidence supporting the therapeutic efficacy of acupuncture. Table 1 summarizes the results of these systematic reviews. A study conducted by Zhao et al. demonstrated that acupuncture had a significant positive impact on various objective sleep measures, such as an increase in total sleep time and sleep efficiency and a decrease in the number of times waking up after falling asleep and the number of arousals. Additionally, subjective sleep quality was also found to improve in patients with PI. The researchers recommended a minimum threshold of at least 12 acupuncture treatments for optimal results (31). Kim et al. analyzed on the duration of acupuncture treatment cycles. Their findings indicated that patients with PI may experience noteworthy symptom improvement throughout acupuncture treatment lasting longer than 3 weeks (32). A comparative analysis was conducted to assess the efficacy of various acupuncture therapies in enhancing Pittsburgh Sleep Quality Index (PSQI) scores. The findings indicated that acupoint catgut embedding, auricular acupressure, or auricular acupuncture combined with manual acupuncture, electroacupuncture combined with acupoint application, and intradermal needle therapy were effective. However, it is important to note that the certainty of the evidence supporting these conclusions was rated as moderate to low (13). Previous research has examined the effectiveness of singular acupuncture stimulation at the SP6 acupoint for addressing insomnia. These studies demonstrated enhancements in sleep quality as well as increases in the duration of both the deep sleep and rapid eye movement phases. However, the systematic review encompassed a limited number of studies, and the overall quality of the literature was deemed to be moderate (33). Consequently, it is imperative to conduct future investigations to scrutinize the findings of the study. According to a meta-analysis conducted by Cao et al., acupuncture demonstrated greater efficacy than pharmacological interventions in terms of increasing the total sleep time by more than 3 h in patients. However, when considering the average sleep duration, there was no discernible distinction between the effects of acupuncture and medication. The combination of acupuncture and medication yielded superior outcomes compared to the administration of medication alone in terms of total sleep duration (15). A study conducted by Kim et al. demonstrated that the application of syndrome differentiation in traditional Chinese medicine acupuncture led to a notable enhancement in the overall effectiveness of treatment compared to that of Western medicine treatments such as nonbenzodiazepine hypnotics and benzodiazepine receptor agonists. However, the improvement in PSQI score was similar between the two approaches (34). A study of insomnia among senior individuals revealed that various interventions, namely, acupuncture, acupuncture in conjunction with benzodiazepines, behavioral treatment, benzodiazepines alone, benzodiazepines combined with CBT, and CBT, produced favorable outcomes. Significantly, the utilization of combination therapy, such as the combination of benzodiazepines with CBT or benzodiazepines with acupuncture, has been found to exhibit overall superiority compared to other forms of single-therapy treatments (35). Hypnotic medications, namely, benzodiazepine agonists, are associated with notable adverse effects, including but not limited to headaches, nightmares, daytime weariness, aberrant behavior, and gastrointestinal disturbances (36). These side effects impose some constraints on the available treatment choices for patients. Previous research has indicated that hematoma, discomfort, headache, and bleeding are the primary mild adverse effects associated with acupuncture (13). These adverse events have been found to pose a relatively low risk for patients with PI.

**Table 1:** Summary of systematic review and meta-analysis results of acupuncture in treating PI.

Comparison	Outcome measures	Findings	Limitations	Refs.
Acupuncture vs. Sham−/placebo-acupuncture or waitlist control	Polysomnography, actigraphy, or micromovement sensitive mattress/pillow sleep monitoring systems	Acupuncture can significantly improve the subjective and objective sleep parameters of PI patients It is suggested that at least 12 times of acupuncture treatment can achieve the best effect	Small research quantity and sample size The strength of the evidence is assessed as low to moderate	(31)
Acupuncture vs. Pharmacotherapy or sham-acupuncture	PSQI, ISI	Compared with drug treatment, after more than 3 weeks of acupuncture treatment, the symptoms of insomnia patients may be significantly improved	Unequal duration of research There are few experiments involving electroacupuncture, and its effectiveness cannot be accurately evaluated and judged	(32)
Acupoint catgut embedding vs. Auricular acupressure or Auricular acupuncture or Electroacupuncture plus Acupoint application.	PSQI, TCM syndrome score, Effective rate	Compared with usual treatment, most types of acupuncture therapy showed the improvementof both subjective and objective sleep indices, especially acupoint catgut embedding, auricular acupressure or auricular acupuncture plus acupuncture, electroacupuncture plus acupoint application	Multiple evidence levels are low It does not find study focused on the potential costs associated with acupuncture interventions	(13)
Warm-acupuncture vs. Acupuncture or Acupoint herbal plaster	PSQI, improvement in clinical effect, PSG	Single acupoint stimulation of SP 6 could improve sleep quality, lengthen deep sleep and REM duration of patients with insomnia	Limited evidence of placebo-controlled trials The reporting and methodological quality of the included trials was low The small numbers of included trials	(33)
Acupuncture vs. Acupuncture plus conventional medication or herbal medicine	PSQI, 12 kinds of scores were used for sleep measurement	Acupuncture appears to be effective in treatment of insomnia. However, further large, rigorous designed trials are warranted	The majority of the included trials were assessed to be of generally fair methodological quality	(15)
Manual acupuncture, Electro-acupuncture vs. Medication	PSQI	Acupuncture using pattern identification led to significantly improved total effectiveness rate compared to medication	High risk of bias, not using a standardized pattern-diagnosis-treatment and not comparing with standarized acupuncture without pattern identification	(34)
Acupuncture vs. acupuncture combined with benzodiazepines or behavioral therapy or benzodiazepines combined with CBT	PSQI	Combined treatments, including benzodiazepines combined with CBT or with acupuncture, were generally superior to other monotherapies	The risk of bias and imprecision of the meta-analyzed results	(35)

PSQI, Pittsburgh sleep quality index; ISI, Insomnia Severity Index; TCM, Traditional Chinese Medicine; PSG, polysomnography; CBT, cognitive behavioral therapy.

In general, acupuncture treatment for PI has produced positive results in many areas, including sleep quality, episodic memory function, daytime drowsiness, attentiveness, stress alleviation, and extended sleep duration, with fewer side effects. However, the quality of the included studies needs to be improved. Therefore, the current evidence suggests that acupuncture alone or combined with drugs for insomnia has a certain therapeutic effect and that the combined treatments can enhance the therapeutic effect of the drug and reduce the required drug dose, thereby reducing adverse reactions.

## 4 Acupuncture types and acupoint selection

**Figure 2:**
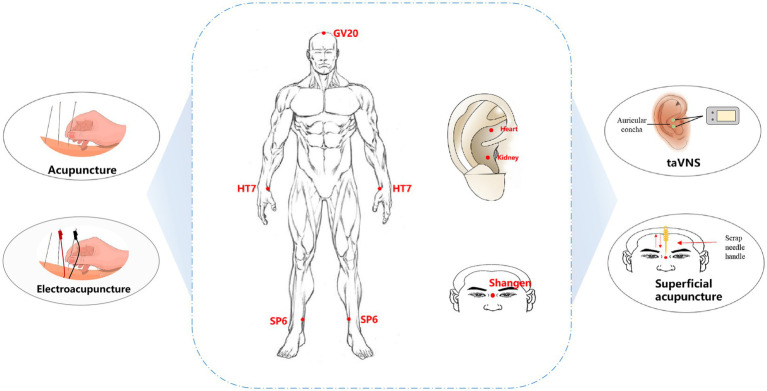
Acupuncture types and acupoint selection for PI treatment. taVNS: transcutaneous auricular vagus nerve stimulation; GV20, Baihui; HT7, Shenmen; SP6, Sanyinjiao.

The growing public fascination with complementary and alternative medicine has led to a heightened focus on the utilization of acupuncture as a potential treatment for insomnia. A comprehensive examination of alternative medicine therapies for insomnia revealed that acupuncture and acupoint massage exhibit promising potential as treatment options for insomnia. These therapies appear to have an impact on the modulation of serotonin, dopamine, and endogenous opioids, which may contribute to their efficacy (37, 38). The acupuncture techniques examined in this study included manual acupuncture, electroacupuncture, transcutaneous auricular vagus nerve stimulation (taVNS), superficial acupuncture, and warm acupuncture (Figure 2), and these approaches elicited therapeutic outcomes by targeting acupoints located on the body’s surface.

Manual acupuncture or electroacupuncture are the most commonly used intervention approaches for PI, and most of the acupuncture manipulations involve mild reinforcing and reducing manipulations. The most frequently used acupoints are HT7 (Shenmen), SP6 (Sanyinjiao), and GV20 (Baihui). These three acupoints are the main recommended points in the Evidence-Based Guideline of Chinese Medicine for Insomnia (Chinese Academy of Chinese Medical Sciences.,2016). HT7 is located at the radial wrist joint of the ulnar flexor muscle at the concave edge of the bean-shaped bone and is considered “the foremost acupoint to calm and regulate the spirit” (39). Multiple meta-analyses have shown that acupuncture is the first choice for treating insomnia and can adjust the imbalance of the core of sleep dysfunction (40, 41). SP6 is the intersection of three Yin meridians (spleen, liver, and kidney meridians from foot to head) and is located three inches above the inner ankle in the depression behind the tibia. A study revealed that stimulating SP6 can increase the content of GABA in the brain, which is similar to benzodiazepine drugs (41). A study on the application patterns of SP6 revealed that it has a wide range of clinical applications and is most commonly used to treat insomnia (42). GV20 is the intersection of the ruling vessel and three yang channels of the hand and foot. It is commonly used in traditional Chinese medicine for the therapeutic management of conditions related to the head, facial features, and mental health disorders (33, 43). TaVNS is also commonly used as a therapeutic approach for PI and involves the combination of traditional auricular acupuncture and vagus nerve stimulation to treat PI by stimulating auricular points located in the vagus nerve region of the ear to modulate the activity of the brainstem, thalamus and cerebral cortex (44). Currently, taVNS is widely used to treat PI, irritable bowel syndrome, depression, and posttraumatic stress disorder (45–47). In the treatment of PI, auricular points are mostly chosen for the heart and kidney regions. According to traditional acupuncture theory, PI is related to heart and kidney disorders, and stimulation of heart and kidney auricular points can regulate the balance of yin-yang and qi-blood in the heart and kidney meridians and improve the clinical symptoms of PI (48). In addition, superficial acupuncture via the Shangen acupoint has been used to treat PI (49). By employing a rhythmic and persistent scraping and pressing motion on the needle handle, subtle and uninterrupted physical vibration is produced, exerting an influence on the Shangen acupoint and eliciting stimulation of the meridian Qi. This stimulation is intended to facilitate sleep and promote a tranquil state of mind.

Notably, in the field of clinical acupuncture, the exact amount of acupuncture stimulation must be mastered to maximize the therapeutic effect. The therapeutic efficacy of acupuncture can be influenced by various factors, including the direction, size, duration, and interval of the needling force. During acupuncture treatment, the patient must provide acupuncture sensory feedback, and any adverse reactions and incidents associated with acupuncture should be documented. Furthermore, comprehensive training must be administered to acupuncturists prior to commencing official experiments to enhance the standardization and uniformity of operational procedures. There were differences in acupuncture parameters among the included studies, indicating the need for large-scale clinical trials to establish standardized operation procedures and acupuncture parameters.

## 5 Effects of acupuncture on brain functional activity in PI patients

The primary pathophysiology of PI is hyperarousal, which leads to alterations in brain regions within the central nervous system that are intimately associated with emotional and cognitive activities in individuals with PI. These alterations are linked to the development, progression, and severity of PI. For instance, sensations of wakefulness can be attributed to alterations in thalamic metabolism (50) and the brainstem reticular arousal system (51, 52).Additionally, impaired subjective sleep quality has been linked to disruptions in hippocampal memory function (53, 54).Currently, various neuroimaging techniques, including functional magnetic resonance imaging (fMRI), electroencephalography (EEG), functional near-infrared spectroscopy (fNIRS), magnetic resonance spectroscopy (MRS), and positron emission tomography (PET), are employed to investigate the underlying central pathogenesis of PI. These techniques offer valuable empirical evidence for studying the pathogenesis and mechanisms of treatment for PI. Recent clinical studies have shown that fMRI, EEG and fNIRS have been used to investigate the effects of acupuncture on functional brain activity in patients with PI, and Table 2 summarizes the characteristics and outcomes of these studies.

**Table 2:** Clinical study on the regulation of brain function activity in primary insomnia by acupuncture.

Neuroimaging technologies	Therapy	Acupoints	Neuroimaging results	Refs
rs-fMRI	MA	EX-HN1, HT7, GV20, SP6	PI (post) vs. HC: *decreased DC value* in MTG, HIP, PUT, PHG, CUN.	(30)
	MA	EX-HN1, GV20, GV16, GB20, GV18, MS13, Anmian, EX-HN13, GV14	PI (post vs. pre): *increased FC* in dorsal CAU to LING, cerebellum; ventral CAU to frontal lobe, INS, ACG; ventral CAU to PreCG, PCG.	(9)
	MA	LI11, ST40, LR3	PI (post vs. pre): *increased ALFF value* in PUT, PAL, CAU; *decreased ALFF value* in CUN, SFG, MFG.	(29)
	MA	GV20, GV24, EX-HN1, HT7, GB13, PC6, SP6	PI (post vs. pre): *decreased FC in* AMYG and THA.	(55)
	EA	Single acupoints group (HT7); multi-acupoints group (HT7, SP6, GV20); Non-acupoint group (the junction point between the biceps brachii muscle and deltoid muscle)	Single acupoints group (post vs. pre): *increased* fALFF *value* in ITG, MTG, STG, PHG, PCUN, SFG; Multi-acupoints group (post vs. pre): *increased* fALFF *value* in PHG, MTG, STG, MFG, IFG, PreCG, PoCG, IPL, SPL, ANG, SMG, PCUN, CUN; Non-acupoint group (post vs. pre): SFG, MFG, IFG, ACG, INS.	(26)
	EA	HT7	Male PI (post vs. pre): *increased* fALFF *value* in MTG, SFG, MFG, PCUN, PHG, SPL, PoCG. Female PI (post vs. pre): *increased* fALFF *value* in MT, PCG, IFG, MFG, STG, SMG.	(56)
	SA	Shangen	PI (post vs. pre): *increased FC* in AMYG to INS, brain stem, CAU, cingulate; *decreased FC* in AMYG to MFG, PCUN.	(57)
	taVNS	Auricular cavum concha	PI (post vs. pre): *increased ReHo value* in SFGmed, ACG; *decreased ReHo value* in ITG, MTG, THA.	(58)
	taVNS	Auricular cavum concha	PI (post vs. pre): *decreased FC* in mPFC to dorsal ACG; *decreased FC* in mPFC to PCUN, CUN, CAL, SOG, LING.	(59)
	taVNS	Auricular cavum concha	Group A (post vs. pre): *decreased* fALFF *value* in cerebellum, SFGmed, SMA.	(46)
	taVNS	Auricular cavum concha	PI (post vs. pre): *decreased FC* in PCG to PCUN, ANG, SFG, MFG, MTG, ORBsupmed; *increased FC* in PCG to LING, CAL.	(60)
	taVNS	Auricular points, heart and kidney areas	Correlation analysis: *ALFF* in SFG, MFG, ACGdor, *fALFF* in IPL, SMG, ANG, *ReHo* in SFG, SMA, ACGdor, PCGdor.	(61)
	taVNS	Auricular points, heart and kidney areas	PI (post vs. pre): *increased FC* in preoptic area to PCUN, PCG, CUN; *decreased FC* in medial THA to PreCG, auxiliary motor area; *increased FC* in medial hypothalamus to PCUN, PCG; *increased FC* in anterior THA to PreCG, SFG, MFG; *increased FC* in anterior beak cingulate gyrus to LING, IOG; *decreased FC* in anterior beak cingulate gyrus to ITG, MTG, IPL, SMG, ANG.	(62)
	taVNS	Auricular points, heart and kidney areas	PI (post vs. pre): *increased FC* in PCG to MFG, PCG to MFG and medial prefrontal lobe.	(59)
	taVNS	Auricular points, heart and kidney areas	PI (post vs. pre): *decreased ALFF* in PCUN, in*creased ALFF* in MOG; *increased FC* in PCUN to ANG, SFG, and MFG.	(63)
	taVNS	Auricular points, heart and kidney areas	PI (post vs. pre): *decreased FC* in THA to ANG, ACG to PCUN.	(64)
task-fMRI	MA	HT7, SP6	PI (post) vs. HC: activated areas included ventral anterior nucleus of THA, putamen, medial globus pallidus, PreCG, CAU.	(65)
	MA	HT7, GV20, SP6	There were differences in brain response areas among the three groups, and the most activated areas included cerebellum, IFG, IPL and MTG.	(66)
	MA	SP6	NOR group: activated areas included STG, IPL, PoCG; SD group: activated areas included ACG, INS, basal ganglia, THA; Sham group: activated areas included THA and cerebellum.	(67)
fNIRS	MA	GV20, PC6, HT7, SP6, KI5	PI (post vs. HC): *decreased HBO concentrations* in frontal primary motor cortex, prefrontal orbitofrontal area, parietal somatosensory association cortex, postero-lateral prefrontal area and premotor cortex.	(68)
EEG	SA	Shangen	PI (post vs. rest): *decreased ApEn* in frontal and occipital lobes, *increased CD* in frontal lobe.	(69)
	SA	Shangen	PI (rest→intervention→post): *ApEn* results for prefrontal lobe →posterior temporal lobe →occipital lobe PI (post vs. rest): *CD* results for frontal, anterior temporal and frontal lobes.	(49)
	MA	BL44, BL47, BL49, BL42, BL52	Observation group (post vs. pre): *decreased* in non-rapid eye movement NREM time, total sleep time; *increased* in sleep efficiency (ratio of actual sleep to total sleep).	(70)
	MA	EX-HN22, PC6, HT7, LI4, ST36, KI6, BL62, LR3	AA (post vs. rest): *the brain electrical potential changes* in the frontal lobe, right temporal lobe, and occipital lobe.	(71)
	taVNS	auricular points, heart, liver and kidney areas	PI (post vs. rest): *increased* in SE, NREM 3, power spectrum of NREM 1-delta1; *decreased* in SL and power spectrum of NREM 1-delta1.	(72)

rs-fMRI, resting-state functional magnetic resonance imaging; task-fMRI, task-based functional magnetic resonance imaging; PI, primary insomnia; HC, healthy control; MA, manual acupuncture; EA, electroacupuncture; taVNS, transcutaneous auricular vagus nerve stimulation; SA, superficial acupuncture; DC, degree centrality; ReHo, regional homogeneity; FC, functional connectivity; ALFF, amplitude of low frequency fluctuation; fALFF, fractional amplitude of low frequency fluctuations; fNIRS, functional near-infrared spectroscopy; EEG, electroencephalogram; EX-HN1, Sishencong; HT7, Shenmen; GV20, Baihui; SP6, Sanyinjiao; GV16, Fengfu; GB20, Fengchi; GV18, Qiangjian; MS13, Dingshangpangxian; EX-HN13, Yuye; GV14, Dazhui; LI11, Quchi; ST40, Fenglong; LR3, Taichong; PC6, Neiguan; KI5, Shuiquan; BL42, Pohu; BL44, Shentang; BL47, Hunmen; BL49, Yishe; BL52, Fuxi; EX-HN22, Jingbi; LI4, Hegu; ST36, Zusanli; KI6, Zhaohai; BL62, Shenmai; MTG, middle temporal gyrus; HIP, hippocampus; PUT, lenticular nucleus, putamen; PHG, parahippocampal; CUN, cuneus; MOG, middle occipital gyrus; SOG, superior occipital gyrus; PCUN, precuneus; PoCG, postcentral gyrus; MFG, middle frontal gyrus; CAU, caudate nucleus; LING, Lingual gyrus; INS, insula; DMN, default mode network; ACG, anterior cingulate and paracingulate gyri; PreCG, precental gyrus; PCG, posterior cingulate gyrus; PAL, lenticular nucleus, pallidum; SFG, superior frontal gyrus; ITG, inferior temporal gyrus; STG, superior temporal gyrus; IFG, inferior frontal gyrus; IPL, inferior parietal, but supramarginal and angular gyri; SPL, superior parietal gyrus; ANG, angular gyrus; SMG, supramarginal gyrus; AMYG, amygdala; SFGmed, superior frontal gyrus, medial; THA, thalamus; mPFC, medial prefrontal cortex; CAL, calcarine fissure and surrounding cortex; SMA, supplementary motor area; ORBsupmed, superior frontal gyrus, medial orbital; ACGdor, anterior cingulate and paracingulate gyri, dorsolateral; PCGdor, posterior cingulate gyrus, dorsolateral; ESS, epworth sleepiness scale; IOG, inferior occipital gyrus; HBO, oxyhemoglobin; ApEn, approximate entropy; CD, correlation dimension; NREM, non-rapid eye movement; SE, sleep efficiency; SL, sleep latency.

### 5.1 Changes in brain functional activity based on neuroimaging technology

In the studies employing fMRI techniques, task-based functional magnetic resonance imaging (task-fMRI) was commonly employed to evaluate the immediate impact of acupuncture, while resting-state functional magnetic resonance imaging (rs-fMRI) was utilized to analyze the cumulative effects of acupuncture. Studies utilizing task-fMRI demonstrated that the administration of acupuncture immediately following treatment can potentially improve insomnia symptoms to a certain degree (55–57). Notably, there are similarities between cumulative acupuncture and immediate acupuncture about the enhancement of brain areas involved in PI. Moreover, the majority of the brain regions that exhibit coordinated activity are associated with cognitive and emotional processes. The investigation of the immediate impact of acupuncture suggested that it may offer valuable clinical guidance for the utilization of acupuncture in patients with PI who have a shorter duration of illness or exhibit minimal symptoms.

Numerous studies have examined the specificity and commonality of acupuncture in the therapeutic application of regularly utilized acupoints for the treatment of PI. In the included studies using rs-fMRI technology, HT7 was the most frequently used acupoint for treating PI. Electroacupuncture of HT7 as a treatment for PI increased the fractional amplitude of low frequency fluctuation (fALFF) values in the parahippocampal gyrus (PHG), superior temporal gyrus (STG), middle temporal gyrus (MTG), superior frontal gyrus (SFG), middle frontal gyrus (MFG), and precuneus (PCUN). The PHG is related to the maintenance of working memory and long-term memory encoding (Schon et al.,2016). The STG and MTG play important roles in attention networks involving emotional regulation and cognitive behavioral processing (58). Abnormal temporal lobe function can cause emotional and cognitive disorders, impulsiveness, poor decision-making, and cognitive regulation disorders (59). The SFG and MFG are believed to be involved in alertness, attention, and higher-order cognitive processes, all of which are disrupted in insomnia patients (60). The function of the PCUN is bidirectional to sleep status. People with increased blood oxygen level-dependent signals in the bilateral anterior cuneiform lobe exhibit greater subjective and objective differences in sleep quality (61), and the functional connectivity density of the anterior cuneiform lobe decreases after sleep deprivation (62). Most of these areas belong to the default mode network (DMN), and the change in fALFF values in these areas after electroacupuncture of HT7 may indicate that PI is treated through the relevant areas of the default network. In a further investigation, acupuncture at the SP6 point elicited more robust and extensive activation of specific brain regions within the saliency network, namely, the anterior cingulate cortex, bilateral insula, left basal ganglia, and thalamus, than sham acupuncture (57). An interesting study compared the effects of single acupuncture (HT7), multiacupuncture combinations (HT7, SP6, GV20), and pseudoacupuncture (junctions between the biceps and deltoids) on PI. These findings indicate that acupuncture is effective at treating PI and that the utilization of a combination of multiple acupoints can enhance its effectiveness. The imaging results showed that the relationship between multipoint combination and brain activity was significantly more regulated than that of single or false acupuncture spots (26). A study conducted by the same research team also investigated potential sex-related variations in the treatment of insomnia with electroacupuncture at HT7. The findings indicated that the PSQI score was significantly lower in women than in men and that disparities related to sex in the effects of electroacupuncture were primarily observed in the posterior cingulate gyrus and supramarginal gyrus (63). These findings offer a neuroimaging-based quantitative foundation for enhancing the understanding of the efficacy of acupoints, as well as their general and unique therapeutic effects.

Research based on fMRI data indicates that acupuncture in treating PI can trigger a series of brain responses. The brain areas primarily implicated in this study included the SFG, MFG, MTG, PHG, PCUN, postcentral gyrus (PoCG), supramarginal gyrus (SMG), and anterior cingulate gyrus (ACG). Among them, the SFG, MTG, PHG, PCUN, SMG, and ACG are important brain regions of the DMN. The relationships between the DMN and emotional processing and episodic memory function have been extensively studied (64). A noteworthy study revealed that individuals diagnosed with PI exhibit disparities in the anterior and posterior subregions of the DMN, impacting cognitive and emotional processing (65, 66). Alterations in functional connectivity within the DMN have an impact on the functioning of both the network itself and the central executive network. Consequently, individuals with PI have symptoms of heightened neural activity, such as excessive daytime drowsiness, weariness, cognitive impairment, and reduced efficiency. The SFG and MFG are located within the frontal lobe and are closely linked to processes related to self-perception and emotional regulation (67). Research has revealed that individuals diagnosed with PI exhibit fewer connections within the right frontoparietal network, specifically in the superior parietal lobule and SFG. These findings suggest a potential association between connectivity reductions and the presence of mood problems in patients suffering from insomnia (68). The PoCG is in the somatosensory cortex, the primary area responsible for receiving tactile input and playing a crucial role in emotional regulation (69). A recent study examined the correlation between sleep quality and functional connectivity within the somatomotor network in a sample of young adult males. The results indicated that individuals with poorer sleep quality, as indicated by higher scores on the PSQI, exhibited heightened functional connectivity within specific regions of the somatomotor network, including the PoCG, left paracentral lobule, bilateral precentral gyrus, and supplementary motor areas. These findings suggest a potential association between sleep quality and the strength of functional connectivity within the somatomotor network in young men (70). Furthermore, a study utilizing fNIRS technology examined the immediate impact of acupuncture on cerebral oxygen saturation in individuals with PI. The findings revealed that acupuncture exerted a positive influence on the functionality of brain regions associated with the frontal and orbitofrontal cortex, as well as the parietal lobe. This effect was achieved through the regulation of oxygenated hemoglobin levels in the cerebral cortex, leading to enhanced control over the involuntary activation of irrelevant stimuli and the modulation of hyperarousal activity. Consequently, acupuncture demonstrated potential benefits in ameliorating insomnia symptoms.

The regulation of the aforementioned brain regions through acupuncture may contribute to the amelioration of difficulties associated with sleep onset, early awakening, excessive daytime sleepiness, fatigue, memory impairment, and reduced cognitive efficiency. This effect is believed to be achieved through the modulation of brain regions involved in cognitive processes, memory formation, emotional regulation, and physical motor functions. The utilization of neuroimaging quantifications, as opposed to relying only on subjective observations, aids in the objective evaluation of the efficacy of acupuncture in treating PI.

### 5.2 Changes in brain functional activity based on EEG technology

The results of studies utilizing EEG techniques have shown that the Shangen acupoint is the most commonly used acupoint, and shallow stimulation of the Shangen acupoint can increase the CD value of the frontal lobe. The frontal cortex is a key component of the pathway for positive and negative emotions (71) while integrating signals from other brain regions that are highly involved in understanding past and current experiences and planning future actions (72). Acupuncture at the Shangen acupoint can enhance frontal lobe brain activity in PI patients, which may help alleviate subjective insomnia and environmental discomfort, thereby reducing bedtime anxiety and contemplation (73). Additionally, acupuncture has the potential to decrease the complexity of EEG signals and exert a significant influence on the electrical activity of the frontal, temporal and occipital lobes (49, 74). The frontal lobe of the brain serves as the primary hub for higher cognitive functions in humans. Additionally, it maintains a significant connection with the regulation of autonomic processes and contributes significantly to autonomous behavior (75). The medial temporal lobe is widely recognized as the “medial temporal lobe memory system,” which specializes in memory function in mammalian brains. This brain region plays a crucial role in the regulation of memory and mood (76). The occipital lobe plays a crucial role in visual processing, making it a significant region for the integration of sensory functions and visual pathways (77). The decreases in approximate entropy and increases in correlation dimension values within the aforementioned brain regions indicate that acupuncture reduces the complexity of PI; makes brain signals more synchronized, stable and orderly; and helps improve difficulty with falling asleep in PI patients. Acupuncture has been found to have the ability to modify the sleep continuity of individuals with PI, manage their sleep structure by decreasing non-rapid eye movement (REM) time and total sleep time, enhance sleep efficiency by increasing the ratio of actual sleep to total sleep, and alleviate the cortical hyperarousal state (78). The findings from these investigations utilizing EEG-based methods established a foundation for elucidating the efficacy and potential mechanisms of acupuncture in treating PI.

## 6 Effects of acupuncture on brain metabolites in PI

Insomnia is strongly correlated with the sleep–wake cycle, which is governed by a diverse range of neurotransmitters and neuromodulators (79). Neurotransmitters can be divided into two primary categories: excitatory and inhibitory neurotransmitters. Acupuncture treatment for PI has been demonstrated to modulate alterations in central neurotransmitter levels that are intimately associated with sleep. This therapeutic approach effectively regulates sleep architecture, enhances sleep promotion, and ultimately improves the overall quality of sleep. Table 3 comprehensively summarizes the key features and results of relevant fundamental research investigating the effect of acupuncture on brain metabolites in experimental animals with PI. Several studies have shown that acupuncture at HT7 and SP6 can increase the 5-HT content in the hypothalamus. Acupuncture at GV20, HT7, and SP6 can increase the 5-HT and MT levels in the hypothalamus, hippocampus, pineal body, and brain while decreasing the NE and DA levels.

**Table 3:** Basic researches on the regulation of brain metabolites in PI by acupuncture.

Detecting brain regions	Experimental animals	Therapy	Acupoints	Biochemical measurements	Refs
Hypothalamus	SD rats	EA	BL13, BL15, BL18, BL20, BL23	5-HT↑, 5-HIAA↑	(92)
	SD rats	EA	HT7, SP6	5-HT↑, Glu↓, GABA↓, Glu/GABA↓	(93)
	Wistar rats	EA	GV20, EX-HN3	Glu↓, Glu/ GABA↓, GABA↑, GABAA↑	(94)
	SD rats	EA	GV20, ST36, SP6	GABA↑	(95)
	Wistar rats	EA	BL13, BL15, BL18, BL20, BL23	5-HT↑, 5-HIAA↑	(96)
	Wistar rats	EA, MA	GV20, EX-HN1	Glu↓, GABA↑	(97)
	SD rats	EA	HT7, SP6, PC6	5-HT↑, GABA↑, Glu↓	(98)
	SD rats	MA	BL62, KI6	Glu↓, GABA↑, Glu /GABA↓, GAD↑, GS↓	(99)
	SD rats	MA	GV20, HT7, SP6	MT↑, MT_1_, MT_2_↑	(100)
	SD rats	MA	GV20, HT7, SP6	MT↑, MT_1_, MT_2_↑	(101)
	SD rats	MA	GV20, HT7, SP6	5-HT_1A_↑, 5-HT_2A_↓	(102)
	SD rats	MA	GV20, HT7, SP6	MT↑	(103)
	Wistar rats	EA, MA	GV20, EX-HN1	5-HT↑, 5-HIAA↑	(104)
	SD rats	MA	HT7, SP6, ST36, PC6, BL62, KI6	GABA↑, GABAR↑	(105)
	SD rats	WA	Dinghui, Heyi, Xin	DA↓	(106)
	Wistar rats	EA	HT7, SP6	5-HT↑, 5-HIAA↑, NE↓	(107)
	C57BL/6 J mice	EA	auricular branch of the vagus nerve	GABA↑	(108)
Hippocampus	Wistar rats	MA	HT7, SP6, ST36, SJ6	5-HT_1A_↑, 5-HT_2A_↓	(109)
	SD rats	WA	Dinghui, Heyi, Xin	NE↓	(110)
	Wistar rats	MA	HT7, SJ6、ST36, SP6	5-HT↑, 5-HIAA↑	(111)
Hypothalamus, hippocampus	SD rats	MA	GV20, HT7, SP6	5-HT↑, NE↓, DA↓	(112)
Brainstem	Wistar rats	EA	GB20, Gongxue	5-HT↑, NE↓, DA↓	(113)
Brainstem	SD rats	WA	GV20, HT7, SP6	5-HT↑, NE↓, DA↓	(114)
Right brain	Wistar rats	MA	HT7, PC6, SP6, ST36, BL62, KI6	5-HT↑	(115)

MA, manual acupuncture; WA, warm acupuncture; EA, electroacupuncture; GV20, Baihui; HT7, Shenmen; SP6, Sanyinjiao; BL13, Feishu; BL15, Xinshu; BL18, Ganshu; BL20, Pishu; BL23, Shenshu; ST36, Zusanli; PC6, Neiguan; BL62, Shenmai; KI6, Zhaohai; EX-HN3, Yintang; EX-HN1, Sishencong; 5-HT, 5-hydroxytryptamine; 5-HIAA, 5-hydroxyindole acetic acid; NE, noradrenaline; DA, dopamine; MT, melatonin; Glu, glutamic acid; GABA, γ-aminobutyric acid; GABAR, γ-aminobutyric acid receptor; GAD, glutamic acid decarboxylase; GS, glutamine synthetase.

In randomized controlled clinical research, the therapeutic efficacy of warm acupuncture combined with auricular acupressure and oral eszolam pills was investigated in patients with PI. The findings of the study demonstrated that both interventions helped enhance sleep quality, with success rates of 83.1 and 87.7%, respectively. However, the use of the encephalofluctuography technique for monitoring alterations in neurotransmitter levels revealed that warm acupuncture combined with auricular acupressure increased the serotonin (5-HT) ratio and the γ-aminobutyric acid/glutamate (GABA/Glu) ratio. Conversely, there was a decrease in norepinephrine (NE) levels. Notably, the administration of oral eszolam tablets did not elicit any changes in neurotransmitter expression within the brain (80).

Previous studies have demonstrated that acupuncture can modulate the expression levels of neurotransmitters in the hypothalamus, hippocampus, and brainstem tissues of rats with chlorophenylalanine-induced insomnia. Specifically, acupuncture has been found to enhance the expression of 5-HT, melatonin (MT), and GABA while reducing the expression of dopamine (DA), NE, Glu, and Glu/GABA. Prior research has indicated that the neurotransmitters 5-HT, DA, and Glu are classified as excitatory neurotransmitters, whereas GABA and NE are classified as inhibitory neurotransmitters. 5-HT is a neurotransmitter that is involved in the sleep–wake mechanism. It plays an important role in the occurrence and maintenance of slow-wave sleep and is considered a “hypnogenic factor” in the brain (81). DA is involved in the regulation of arousal and the modulation of behavioral excitement (82). Glu serves as the primary excitatory neurotransmitter in the central nervous system, facilitating neuronal communication, while GABA neurons function as inhibitory agents, regulating neural activity. The Glu/GABA ratio is considered a reliable measure for assessing the balance between excitation and inhibition in nerve cells, providing valuable insights into the therapeutic efficacy of insomnia treatment (83). NE primarily influences the regulation of REM sleep and wakefulness, exhibiting both excitatory and inhibitory impacts on central neurons. Notably, NE plays distinct roles in particular regions (84). Furthermore, MT is a prominent hormone that is actively secreted by the pineal gland in mammals. The secretion of MT is intricately linked to light exposure, and it plays a crucial role in regulating the circadian rhythm of sleep and influencing the overall quality of sleep (85). Acupuncture modulates these neurotransmitters to enhance the sleep–wake cycle and ameliorate the quality of sleep.

Simultaneously, the researchers conducted basic experiments to investigate the optimal electroacupuncture stimulation parameters for treating insomnia in rats. Their findings indicated that the sparse wave (10 Hz) had a superior effect compared to the dense wave (50 Hz) and the combined sparse wave (10/50 Hz) (86, 87). The efficacy of a stimulation frequency of 2 Hz was found to be superior to that of frequencies of 50 Hz and 100 Hz (88). Furthermore, a stimulation intensity of 2 V was more effective than 1 V (87), and the immediate acupuncture effect lasted more than 12 h. The results of this study could lead to the development of clinical electroacupuncture interventions. Low-frequency electroacupuncture stimulation may be more suited for modulating the sleep–wake cycle to induce relaxing and hypnotic impacts.

## 7 Other effects of acupuncture in the brain on PI

The sleep–wake cycle can be influenced by various mechanisms in the brain, such as circadian clock genes, immunological components, energy metabolism, and apoptosis. Several researchers have also examined the potential of acupuncture to enhance sleep quality through the regulation of these parameters. Table 4 presents a comprehensive overview of the key attributes and outcomes derived from existing fundamental investigations about the neurological impacts of acupuncture in treating PI.

**Table 4:** Basic researches on acupuncture regulating other effects of PI in the brain.

Detecting brain regions	Experimental animals	Therapy	Acupoints	Biochemical measurements	Refs
Hypothalamus	SD rats	WA	Dinghui, Heyi, Xin	PRELP, NSMF, TMEM41B, MAP1B	(123)
	SD rats	EA	HT7, SP6	AMPK↓, Ac-CoA↑, NA^+^-K^+^-ATP↑	(124)
	SD rats	EA	BL13, BL15, BL18, BL20, BL23	TNF-α↑, IL-1β↑	(92)
	SD rats	MA	BL62, KI6	Per1↑, Per2↑	(125)
	SD rats	MA	GV20, HT7, SP6	Bmal1↑, Clock↑	(126)
	Wistar rats	MA, EA	GV20, EX-HN1	Bmal1↑, Clock↑, Per1↑	(97)
	SD rats	MA	BL62, KI6	Clock↑, Per2↑	(99)
	SD rats	MA	HT7, LR3, BL15, BL18	P38 MAPK↓	(127)
Hippocampus, brainstem	SD rats	MA	GV20, HT7, SP6	Bcl-2↑, Bax↓, Bad↓, Caspase-3↓, TrkB↑, p-TrkB↓, PI3K↑, p-Akt↑	(112)
Hippocampus	SD rats	EA	GV20, HT7, SP6	PKA-Cβ↑, p-CREB↑, BDNF↑, TrkB↑	(128)
Brain	SD rats	MA	BL15, BL18, BL20, BLl3, BL23	PI3K↑, Akt	(129)
	Wistar rats	MA	GV20, PC6, HT7, LR3	blood oxygen metabolism in the prefrontal and occipital lobes of the cerebral cortex↑	(130)

MA, manual acupuncture; WA, warm acupuncture; EA, electroacupuncture; GV20, Baihui; HT7, Shenmen; SP6, Sanyinjiao; BL13, Feihu; BL15, Xinshu; BL18,Ganshu; BL20, Pishu; BL23, Shenshu; BL62, Shenmai; KI6, Zhaohai; EX-HN1, Sishencong; PRELP, prolargin; NSMF, NMDA receptor synaptonuclear-signaling and neuronal migration factor; TMEM41B, transmembrane protein 41B; MAP1B, microtubule-associated protein 1B; PBR, peripheral benzodiazepine receptor; TrkB, tropomysin related kinase B; PI3K, phosphoinositide 3-kinase; Akt, protein kinase B; AMPK, adenosine 5′-monophosphate (AMP)-activated protein kinase; Ac-CoA, acetyl-coenzyme A; ATP, adenosine triphosphate; TNF-α, tumour necrosis factor alpha; IL-1β, interleukin-1beta; Per1, period1; Bmal1, bain and muscle arnt-like protein-1; Clock, circadian locomotor output cycles kaput; PKA-Cβ, catalytic subunit of protein kinase A; p-CREB, phosphorylated cAMP-responsive element-binding protein; BDNF, brain-derived neurotrophic factor.

Insufficient sleep or dysrhythmia can have a direct impact on the circadian clock genes responsible for regulating the operation of circadian rhythms in the brain. Disruption of the circadian rhythm motor output cycle failure (clock)–brain and muscle aromatic hydrocarbon receptor nuclear transport-like 1 (bmal1)-related pathway (89) leads to noticeable inflammatory effects characterized by an imbalance in the ratio of proinflammatory factors to anti-inflammatory factors (90, 91). Circadian rhythm problems have been found to potentially induce apoptosis of neuronal cells in the brain through the involvement of the Bcl-2 family and caspase family (92). Additionally, these disorders can impact mitochondrial energy metabolism via the AMPK signaling pathway (93). Furthermore, alterations in the mitochondrial membrane potential might further contribute to the exacerbation of apoptosis. The hypothalamus is a crucial brain region involved in the regulation of sleep, energy metabolism, and the immune response. Within this region, the paraventricular nucleus plays a significant role in the regulation of arousal, and the suprachiasmatic nucleus serves as the primary circadian “pacemaker” in the mammalian brain (94, 95). Consequently, the hypothalamus is predominantly utilized by researchers for observation.

In studies conducted by Wei, Guo, Xing, and Liu, acupuncture was shown to increase the expression levels of circadian clock genes (Clock, Bmal1, and recombinant period circadian protein (Per)) in the hypothalamus of rats with insomnia. This upregulation of gene expression was found to enhance rhythms of spontaneous activity and facilitate the restoration of the disrupted sleep–wake cycle (96–98). Tang’s study demonstrated that the application of electroacupuncture at the Wushu point effectively modulated the release of neurotransmitters, specifically 5-HT and 5-HIAA, through the upregulation of the proinflammatory factors TNF-α, L-1β and P38 MAPK in the hypothalamus of insomnia rats. This mechanism ultimately led to the amelioration of pathological damage in hippocampal tissues and the improvement of insomnia symptoms in the experimental rats (99). In a study conducted by Zheng, acupuncture administered at the HT7 and SP6 acupoints in rats with insomnia resulted in a notable decrease in the expression of AMPK, a receptor involved in cellular energy regulation within the hypothalamus. Conversely, there was a large increase in the levels of Ac-CoA and ATP generated through energy metabolism. These findings suggest that acupuncture has the potential to exert a positive regulatory influence on energy metabolism, leading to notable improvements in insomnia and feelings of exhaustion after experiencing insomnia (100). In the study conducted by Cao, the focus was on investigating the regulatory mechanism of acupuncture on insomnia in rats. Specifically, this study examined the impact of acupuncture on apoptosis and identified the effects of acupuncture at specific acupoints, namely, GV20, HT7, and SP6. Acupuncture at these acupoints can increase the expression of Bcl-2; reduce the expression of Bax, Bad and Caspase-3; and reduce the apoptosis of cells in the brain by regulating the expression of proteins related to the PI3K/AKT signaling pathway (101). Furthermore, Xu employed proteomics technology to investigate the proteomic alterations induced by acupuncture treatment in the hypothalamus of rats with insomnia. The findings revealed that the therapeutic impact of acupuncture primarily involved the modulation of four distinct proteins associated with the repair of nerve injuries (PRELP, NSMF, TMEM41B, and MAP1B). A new study has explored the potential mechanism of acupuncture in improving spatial learning and memory deficits in a rat model of PI. The research results found that electroacupuncture with GV20, HT7, and SP6 can upregulate PKA/CREB and BDNF/TrkB signaling in the hippocampus of PI rats, regulate hippocampal neural plasticity, inhibit neuronal apoptosis, and exert a synergistic effect on improving spatial learning and memory impairment (102). These results provide additional evidence suggesting that acupuncture can restore neurodevelopment and may serve as a crucial therapeutic approach for effectively alleviating symptoms of insomnia (103). In addition, basic research has also found similar results to clinical research, suggesting that acupuncture may improve abnormal behavior in insomnia rats by regulating blood oxygen metabolism in the prefrontal and occipital lobes of the cerebral cortex (104).

## 8 Limitations and perspectives for future studies

This study has certain limitations. The included studies were all in Chinese and English, so there may be a language bias. In terms of clinical research, this review explored the role of acupuncture and the factors that affect acupuncture efficacy from multiple perspectives. However, several clinical trials had small sample sizes, which might have contributed to larger random errors and limited statistical power. Some existing trials lack unified quantitative indicators of acupuncture treatment (acupuncture depth, stimulation intensity, acupuncture angle, treatment cycle, stimulation duration, etc.). To improve the repeatability and comparability of research findings, future studies should strive to develop standardized acupuncture operation procedures (105). “*Deqi*” is a key factor in the effectiveness of acupuncture treatment, but most of the included studies lack a description of the sensation and intensity of “*Deqi*.” Future studies should combine the standardized assessment of the “*Deqi*” sensation, which will also provide valuable insights into its therapeutic role. To further study the complex mechanism of acupuncture and improve the quality of experimental results, future research can use multimodal neuroimaging technology and diversified analysis methods (106), which will help to more comprehensively and carefully understand the neural changes caused by acupuncture. In addition, given the scattered entry points of current basic research and poor correlation, most studies mainly focus on the level of neurotransmitters, and the effect of acupuncture is the result of the joint action of multiple systems. Therefore, a systematic research plan integrating multiple systems, organs and targets is needed, which will provide an overall perspective on the effect of acupuncture in treating insomnia, and help deepen the understanding of the underlying central nervous system mechanism of acupuncture in treating insomnia.

## 9 Conclusion

**Figure 3:**
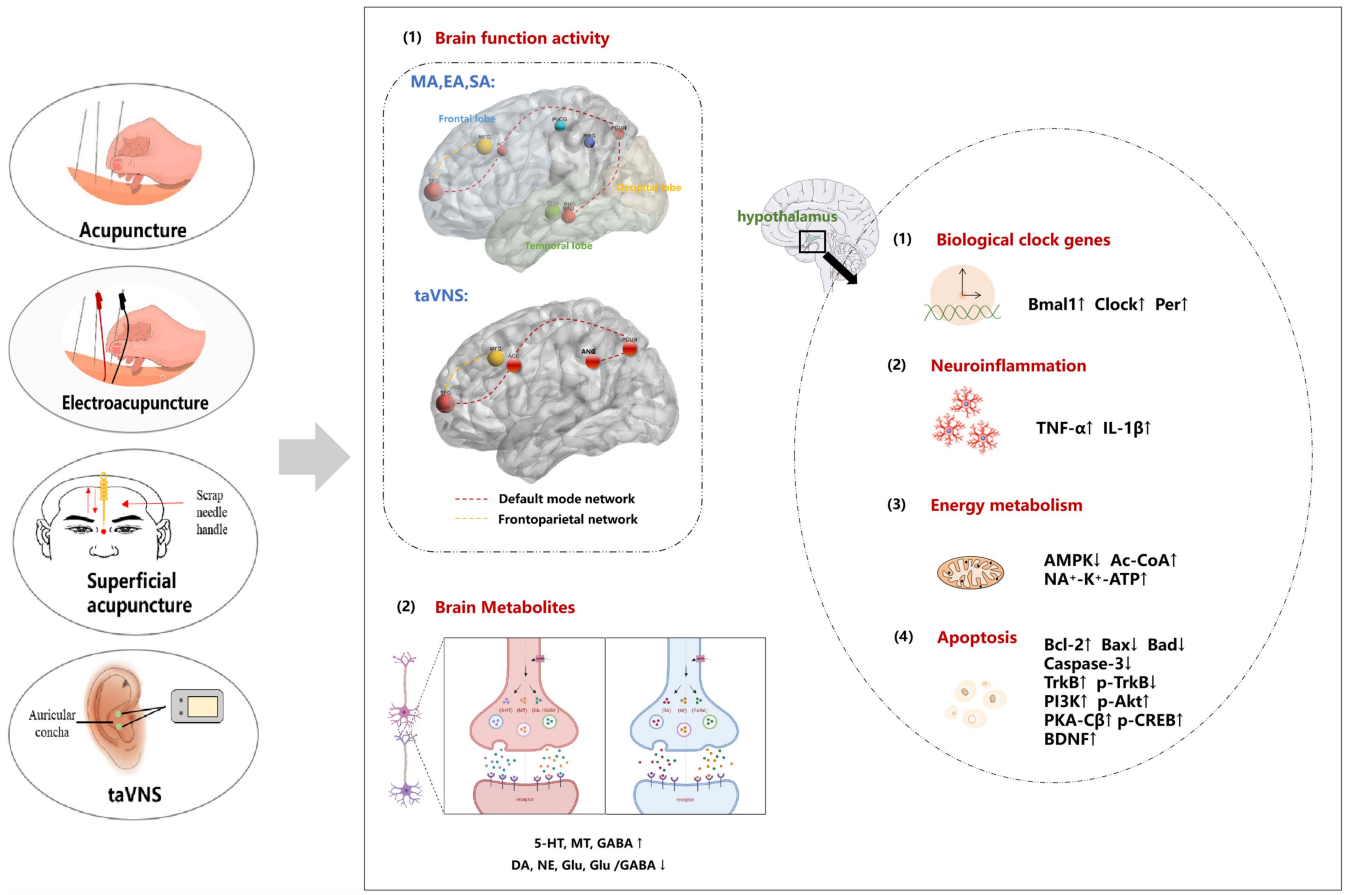
The potential central regulatory mechanism of acupuncture for PI. (1) Acupuncture improves brain function by regulating brain regions such as the default network and frontal lobe network. (2) Acupuncture improves the sleep–wake cycle by increasing the expression of neurotransmitters such as 5-HT, MT and GABA and reducing the expression of DA NE, and Glu and the Glu/GABA ratio. (3) Acupuncture affects sleep rhythm by regulating the expression of circadian clock genes, specifically by upregulating the clock, Bmal1, and Per genes. () The therapeutic effects of acupuncture in relieving brain tissue damage involve the promotion of inflammatory factor synthesis and modulation of neurotransmitter release. (5) Acupuncture influences energy metabolism by regulating AMPK-related pathways. (6) The neuroprotective effects of acupuncture are attributed to its ability to inhibit neuronal apoptosis through the regulation of the PI3K/AKT, PKA/CREB or BDNF/TrkB signaling pathway, thereby restoring nerve function. MA, manual acupuncture; EA, electroacupuncture; taVNS, transcutaneous auricular vagus nerve stimulation; SA, superficial acupuncture; 5-HT, 5-hydroxytryptamine; NE, noradrenaline; DA, dopamine; MT, melatonin; Glu, glutamic acid; GABA, γ-aminobutyric acid; Per1, period1; Bmal1, bain and muscle arnt-like protein-1; Clock, circadian locomotor output cycles kaput; TNF-α, tumour necrosis factor alpha; IL-1β, interleukin-1beta; AMPK, adenosine 5′-monophosphate (AMP)-activated protein kinase; Ac-CoA, acetyl-coenzyme A; ATP, adenosine triphosphate; TrkB, tropomysin related kinase B; PI3K, phosphoinositide 3-kinase; Akt, protein kinase B; PKA-Cβ, catalytic subunit of protein kinase A; p-CREB, phosphorylated cAMP-responsive element-binding protein; BDNF, brain-derived neurotrophic factor.

Previous studies have confirmed the efficacy of acupuncture in resolving PI status, improving the sleep quality of PI patients, prolonging sleep time and minimizing adverse consequences. The combination of acupuncture with drug therapy has been proven to improve the therapeutic efficacy of drugs while reducing the required dose and related adverse reactions. The results of this study showed that acupuncture can regulate the neural function network in the brain, the expression of neurotransmitters, the inflammatory reaction and the energy metabolism of the peripheral immune system; inhibit apoptosis; improve sleep rhythm and sleep quality; and alleviate emotional memory dysfunction caused by insomnia (Figure 3 for details). In addition, the regulatory effects of single acupuncture and cumulative acupuncture on brain functional areas are similar. Acupuncture, as a feasible nondrug intervention measure, has considerable potential for the clinical management of PI. This treatment method demonstrates the ability not only to improve the quality of sleep in patients but also to regulate the emotional memory dysfunction caused by insomnia. The results of this study can provide a reference for basic research on acupuncture and will also provide theoretical guidance for clinical research.
