# Supplementary material for: The central regulatory effects of acupuncture in treating primary insomnia: a review
Source: Front Neurol. 2024 Dec 10;15:1406485. doi: 10.3389/fneur.2024.1406485 (PMC11666528; doi:10.3389/fneur.2024.1406485)
Supplement: Supplementary file 1 [file Data_Sheet_1.docx]

**Pubmed**

| Number | Search term |
| --- | --- |
| #1 | Sleep Initiation and Maintenance Disorders [Mesh] |
| #2 | Disorders of Initiating and Maintaining Sleep [ti,ab] OR DIMS (Disorders of Initiating and Maintaining Sleep) [ti,ab] OR Early Awakening [ti,ab] OR Awakening, Early [ti,ab] OR Nonorganic Insomnia [ti,ab] OR Insomnia, Nonorganic [ti,ab] OR Primary Insomnia [ti,ab] OR Insomnia, Primary [ti,ab] OR Transient Insomnia [ti,ab] OR Insomnia, Transient [ti,ab] OR Rebound Insomnia [ti,ab] OR Insomnia, Rebound [ti,ab] OR Secondary Insomnia [ti,ab] OR Insomnia, Secondary [ti,ab] OR Sleep Initiation Dysfunction [ti,ab] OR Dysfunction, Sleep Initiation [ti,ab] OR Dysfunctions, Sleep Initiation [ti,ab] OR Sleep Initiation Dysfunctions [ti,ab] OR Sleeplessness [ti,ab] OR Insomnia Disorder [ti,ab] OR Insomnia Disorders [ti,ab] OR Insomnia [ti,ab] OR Insomnias [ti,ab] OR Chronic Insomnia [ti,ab] OR Insomnia, Chronic [ti,ab] OR Psychophysiological Insomnia [ti,ab] OR Insomnia, Psychophysiological [ti,ab] |
| #3 | (#1 OR #2) |
| #4 | Acupuncture [Mesh] |
| #5 | Acupuncture therapy [ti, ab] OR Acupuncture, Ear [ti, ab] OR Auricular Acupuncture [ti, ab] OR Acupuncture points [ti, ab] OR Needle [ti, ab] OR Manual Acupuncture [ti, ab] OR Electroacupuncture [ti, ab] OR Laser Acupuncture [ti, ab] OR Acupressure [ti, ab] OR OR Scalp Acupuncture [ti, ab] OR Warm Acupuncture [ti, ab] OR Skin Acupuncture [ti, ab] OR Acupoints [ti, ab] |
| #6 | (#4 OR #5) |
| #7 | Brain [ti, ab] OR central nervous system [ti, ab] |
| #8 | (#3 AND #6 AND#8) |

**Web of science**

| Number | Search term |
| --- | --- |
| #1 | (((((((((((((((((((((((((((TS=(Sleep Initiation and Maintenance Disorders)) OR TS=(Disorders of Initiating and Maintaining Sleep)) OR TS=(DIMS (Disorders of Initiating and Maintaining Sleep))) OR TS=(Early Awakening)) OR TS=(Awakening, Early)) OR TS=(Nonorganic Insomnia)) OR TS=(Insomnia, Nonorganic)) OR TS=(Primary Insomnia)) OR TS=(Insomnia, Primary)) OR TS=(Transient Insomnia)) OR TS=(Insomnia, Transient)) OR TS=(Rebound Insomnia)) OR TS=( Insomnia, Rebound)) OR TS=(Secondary Insomnia)) OR TS=(Insomnia, Secondary)) OR TS=(Sleep Initiation Dysfunction)) OR TS=(Dysfunction, Sleep Initiation)) OR TS=(Dysfunctions, Sleep Initiation )) OR TS=(Sleep Initiation Dysfunctions)) OR TS=(Sleeplessness)) OR TS=(Insomnia Disorder)) OR TS=(Insomnia Disorders)) OR TS=(Insomnia)) OR TS=(Insomnias)) OR TS=(Chronic Insomnia )) OR TS=(Insomnia, Chronic)) OR TS=(Psychophysiological Insomnia)) OR TS=(Insomnia, Psychophysiological) |
| #2 | ((((((((((((((TS=(Acupuncture)) OR TS=(Acupuncture therapy)) OR TS=(Acupuncture, Ear)) OR TS=(Auricular Acupuncture)) OR TS=(Acupuncture points)) OR TS=(Needle)) OR TS=(Manual Acupuncture)) OR TS=(Electroacupuncture)) OR TS=(Laser Acupuncture)) OR TS=(Acupressure)) OR ) OR TS=(Scalp Acupuncture)) OR TS=(Warm Acupuncture)) OR TS=(Skin Acupuncture)) OR TS=(Acupoints)) OR TS=(Acupoints) |
| #3 | ((TS=(Brain)) OR TS=(central nervous system)) |
| #4 | (#1 AND #2 AND #3) |

**China National Knowledge Infrastructure**

| #1 | SU=(失眠+失眠症+原发性失眠+顽固性失眠+入睡困难+睡眠障碍+入睡和睡眠障碍+睡眠不足+不寐+不得寐) AND SU=(针刺+针灸+电针+火针+温针+手针+体针+耳针+皮肤针) AND SU=(脑+中枢) |
| --- | --- |

**Wangfang Data**

| #1 | 主题：("失眠" or "失眠症" or "原发性失眠" or "顽固性失眠" or "入睡困难" or "睡眠障碍" or "入睡和睡眠障碍" or "睡眠不足" or "不寐" or "不得寐") and ("针刺" or "针灸" or "电针" or "火针" or "温针" or "手针" or "体针" or "耳针" or "皮肤针") and ("脑" or "中枢") |
| --- | --- |
